# Resistance training preserves high-intensity interval training induced improvements in skeletal muscle capillarization of healthy old men: a randomized controlled trial

**DOI:** 10.1038/s41598-020-63490-x

**Published:** 2020-04-20

**Authors:** Aurel B. Leuchtmann, Sandro Manuel Mueller, David Aguayo, Jens A. Petersen, Maria Ligon-Auer, Martin Flück, Hans H. Jung, Marco Toigo

**Affiliations:** 1 Institute of Translational Medicine ITM, ETH Zurich, Zurich, Switzerland; 2 Department of Neurology, University Hospital Zurich, Zurich, Switzerland; 3 Institute of Human Movement Sciences, ETH Zurich, Zurich, Switzerland; 4 Research and Performance Center for Elite Athleticism, OYM, Cham, Switzerland; 5 Laboratory for Muscle Plasticity, Balgrist University Hospital, Department of Orthopaedics, University of Zurich, Zurich, Switzerland; 6 Present Address: Biozentrum, University of Basel, Klingelbergstrasse 50/70, CH-4056 Basel, Switzerland; 7 Present Address: Kieser Training, Zurich, Switzerland

**Keywords:** Ageing, Risk factors

## Abstract

Skeletal muscle capillarization is a determining factor in gas and metabolite exchange, while its impairments may contribute to the development of sarcopenia. Studies on the potential of resistance training (RT) to induce angiogenesis in older muscles have been inconclusive, and effects of sequential endurance training (ET) and RT on capillarization are unknown. Healthy older men (66.5 ± 3.8 years) were engaged in either 12 weeks of habitual course observation (HC) followed by 12 weeks of RT (*n* = 8), or 12 weeks of high-intensity interval training (HIIT) followed by 12 weeks of RT (*n* = 9). At baseline, following 12 and 24 weeks, *m. vastus lateralis* biopsies were obtained. (Immuno-)histochemistry was used to assess indices of muscle fiber capillarization, muscle fiber morphology and succinate dehydrogenase (SDH) activity. Single periods of RT and HIIT resulted in similar improvements in capillarization and SDH activity. During RT following HIIT, improved capillarization and SDH activity, as well as muscle fiber morphology remained unchanged. The applied RT and HIIT protocols were thus similarly effective in enhancing capillarization and oxidative enzyme activity and RT effectively preserved HIIT-induced adaptations of these parameters. Hence, both, RT and HIIT, are valid training modalities for older men to improve skeletal muscle vascularization.

## Introduction

Aging is associated with reduced cardiorespiratory fitness^1,2^ and impaired responsiveness of skeletal muscle tissue to anabolic stimuli^3–5^. Skeletal muscle capillarization can affect both cardiorespiratory fitness and muscle anabolism. Enhanced capillarization ameliorates gas and metabolite exchange between blood and muscle tissue, resulting in improved peak oxygen uptake ($\dot{V}{O}_{2peak}$) and oxidative capacity^6,7^. Likewise, it has been proposed, that increased blood flow to muscles is required to support muscle fiber hypertrophy and satellite cell activation in older adults by facilitating the delivery of nutrients, cytokines and growth factors^8,9^. Furthermore, a recent study by Prior *et al*.^10^ revealed that sarcopenic older adults have lower values for capillarization indices when compared to non-sarcopenic controls. Taken together, emerging evidence suggests that a decline in capillarization with increasing age might contribute to the development of sarcopenia and functional impairments in older adults.

Endurance-type training (ET) is considered the method of choice for improving capillarization in skeletal muscle and inducing the associated local and systemic health-related benefits. While ET has consistently been demonstrated to stimulate angiogenesis and to substantially increase skeletal muscle capillarization and $\dot{V}{O}_{2peak}$ in older adults^6,7,11^, data on the potential of resistance training (RT) to effectively induce angiogenesis in aged muscle has been inconclusive. Some studies reported an increase in capillarization following 9-24 weeks of RT^6,9,11,12^, but others observed no effect of RT on capillarization indices^8,13,14^. However, most of the studies investigating the effect of training on capillarization in older adults, considered either RT or ET groups, and did not include both training modalities. Consequently, it is not possible to infer from these studies whether there are adaptive differences of capillarization indices between the two training modalities.

To elucidate whether RT and ET differ in their effects on structural adaptations, the inclusion of both training modalities into one study together with a sequential training design is required. So far, only one study has investigated the effects of sequential RT and ET on capillarization^6^. The authors observed a similar increase in capillarization indices after 18 weeks of ET compared to 9 weeks of RT followed by 9 weeks of ET. More importantly, the increased capillary-to-fiber ratio and number of capillary contacts after 9 weeks of RT could not be further increased by the following 9 weeks of ET. These findings indicate that the two distinct and sequentially applied exercise modalities do not differ in their potential to increase capillarization indices in older adults. Furthermore, there appears to be a mechanistic overlap in the underlying adaptation processes for the studied population, as the subsequent ET did not act synergistically to further increase capillary supply. However, the reverse sequence, in which a period of ET precedes a period of RT, has not been investigated so far.

In this study, we aimed at investigating adaptation patterns of capillarization indices in study groups that were subjected to single periods of exclusive RT or ET in the form of high-intensity interval training (HIIT). We have chosen HIIT as the type of ET, because a solid body of evidence suggests that HIIT has similar if not superior effects on parameters quantifying ET adaptations, while being well tolerated even in diseased populations^15^. Importantly, compared to traditional endurance-based training, HIIT appears to be more time efficient and is generally perceived as less monotonous, which clearly helps to overcome issues with adherence and compliance. To further characterize possible adaptation differences between RT and ET, we imposed study groups to a period of RT without prior training or subsequent to a period of HIIT and compared capillarization indices.

## Methods

### Participants

Twenty older recreationally active men, recruited from the area of Zurich (Switzerland), voluntarily participated in this study. Participants were free of any musculoskeletal or other disorders that could potentially affect their ability to complete testing and/or training. They had no experience with systematic RT or ET for the previous 2 years prior to their enrolment. Two participants withdrew from the study for personal reasons not related to the study. An additional participant was excluded from the final analyses, because he did not fulfill the training frequency guidelines. In total, 17 participants (66.5 ± 3.8 years, 82.4 ± 13.0 kg, 177 ± 5 cm, 2.28 ± 0.37 W·kg^−1^) completed the training intervention and all assessments. Prior to enrolment in the study, participants were fully informed about the purposes, benefits and risks associated with the study and completed a routine health questionnaire before giving written informed consent to their participation in the study. In case of a health-related doubt, consent of a physician was obtained. The study was approved by the ethics committee of the Canton of Zurich [Kantonale Ethik-Kommission Zürich (KEK) reference number: KEK-ZH-Nr. 2013-0114] and was conducted in accordance with the ethical standards laid down in the Declaration of Helsinki for human experimentation. The trial was registered at clinicaltrials.gov as NCT 01905345 (date of registration: 23/07/2013). Participants were recruited between 01/08/2013 and 31/12/2015 and the trial ended at 01/08/2017 because of low accrual rate.

### Study design

Participants were randomly assigned to one of two groups by drawing lots: 12 weeks of a habitual course observation phase followed by 12 weeks of progressive RT (HC→RT, *n* = 8) or 12 weeks of HIIT followed by 12 weeks of progressive RT (HIIT→RT, *n* = 9). Participants were instructed to maintain their individual physical activity level and were advised not to include other types of systematic training or additional physical activities, which they were not already practicing before their enrolment. Before, between, and after the two 12-week phases of the study, a percutaneous muscle biopsy was obtained from the *m. vastus lateralis*.

### Training regimen

Participants performed three training sessions per week at the Institute of Human Movement Sciences, Exercise Physiology, University of Zurich Irchel. Training adherence was 85% on average. All training sessions were supervised by a movement scientist. During the training sessions, participants were verbally encouraged by the supervisor. After each HIIT and RT session, participants received a drink containing 30 g of whey protein (Scientifics, Schwyz, Switzerland) in order to reduce variation in the intake of post-exercise protein and thereby the immediate anabolic response to the exercise training.

### High-intensity interval training (HIIT)

A HIIT session started with a 3-min warm-up at 50% peak power (of a previously conducted incremental cycling test) on a bicycle ergometer (Bike XT, Technogym, Gambettola, Italy). Subsequently, power was increased to 65% peak power for 4 min. Afterwards, participants conducted seven 1-min high-intensity intervals at 85% peak power. Between the high-intensity intervals, power was reduced to 65% peak power for 4 min each time. As soon as a participant successfully completed the whole training session at the target values, power was increased by 3% peak power in the subsequent training session. The applied protocol was adopted from Charifi *et al*.^16^, using minor modifications to make the protocol more strenuous: 65% peak power instead of 65–75% $\dot{V}{O}_{2peak}$ and 85% peak power instead of 85–95% $\dot{V}{O}_{2peak}$.

### Resistance training (RT)

During RT, three exercises (leg extension, leg press, and squat) were conducted under supervision, assuring anatomically correct techniques. The participants performed three sets of each exercise with a 3-min resting period between sets. Every set was performed to volitional muscular failure. One repetition consisted of a 4-s shortening action, a 2-s isometric transition phase and a 4-s lengthening action. Each repetition had to be performed over the individual’s full range of motion. In the first training session, participants started with approximately 60% of their previously determined 1-repetition maximum. In subsequent training sessions, the load was increased by the smallest possible unit, whenever participants exceeded a time under tension (TUT) of 120 s per set. For the leg extension exercise, an average TUT per set of 103 ± 8 s for the HC→RT and 106 ± 10 s for the HIIT→RT group was accomplished. For the leg press exercise, a mean TUT per set of 112 ± 15 s and 113 ± 10 s was achieved for the HC→RT and HIIT→RT group, respectively. The squat exercise resulted in a TUT per set of 103 ± 12 s and 102 ± 11 s for the HC→RT and HIIT→RT group, respectively. Between RT sessions, participants rested for at least 48 h.

### Muscle biopsy sampling

Muscle biopsies were taken at the University Hospital Zurich, Division of Neurology by an experienced physician. All biopsies were obtained from the *m. vastus lateralis* of the dominant leg after local anesthesia with 1% lidocaine, using a 6-mm Bergström needle (Dixons Surgical Instruments, Essex, UK) with suction applied. Biopsies served for the quantification of skeletal muscle capillarization and oxidative enzyme activity. All participants were instructed to refrain from exercise for 24 h before and 72 h after each biopsy. Initial biopsies were obtained from the mid-portion of the *m. vastus lateralis*, ~18 cm proximal to the patella, approximating the midline of the quadriceps muscle group. The two subsequent biopsy procedures were performed at a distance of 1-2 cm proximal or distal to the site of the first biopsy in order to minimize effects of previous incisions. Muscle samples were immediately mounted in an embedding medium (Tissue-Tek, Sakura, Zoeterwoude, The Netherlands), snap frozen in nitrogen-cooled isopentane and subsequently stored at −80 °C until use.

### Histochemistry

The 12 µm cryocut cross-sections were enzyme-immunohistochemically stained using the monoclonal mouse anti-human CD31 endothelial antibody (DAKO, Carpinteria, CA, USA, 1:600) as marker for muscle capillaries, a VECTASTAIN^®^ Elite^®^ABC-Peroxidase Kit (Mouse IgG; Vector laboratories, Burlingame, CA, USA) and Liquid DAB+ Substrate Chromogen as peroxidase substrate (DAKO, Carpinteria, CA, USA, 1:50). Afterwards, sections were rinsed in tap water, counterstained in 0.1% Eosin Y (Sigma-Aldrich, Saint Louis, MO, USA), dried and mounted with Thermo Scientific™ Immu-Mount™ (Thermo Fisher Scientific, Pittsburgh, PA, USA). For estimating muscle fiber oxidative capacity, sections were stained for succinate dehydrogenase (SDH) activity in sarcosomes. Thawed sections were incubated in phosphate buffered media (pH 7.6) containing the SDH substrate succinate in the form of succinic acid (Fluka Chemie, Buchs, Switzerland) and nitro blue tetrazolium chloride (Merck Millipore, Darmstadt, Germany) at 37 °C. Upon incubation, succinate is oxidized to fumarate by SDH revealing free hydrogen atoms, which in turn are used to reduce nitro blue tetrazolium to nitro blue diformazan. Nitro blue diformazan is insoluble in aqueous solutions and precipitates to purple aggregates at the place where it is formed. After incubation in media for 1.5-5 h at 37 °C until desired stain intensity developed, sections were washed in PBS for 5 min, dried and mounted with Thermo Scientific™ Immu-Mount™ (Thermo Fisher Scientific, Pittsburgh, PA, USA).

### Muscle biopsy analyses

**Figure 1:**
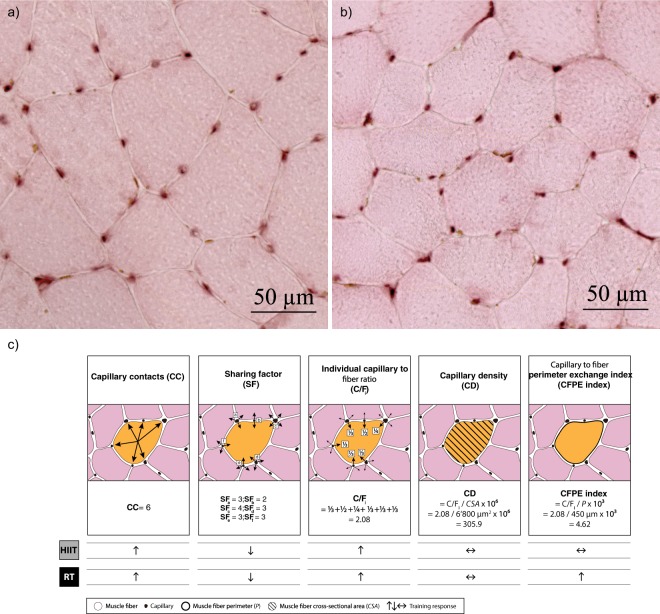
Representative images for the CD31 staining in the (**a**) HC→RT and (**b**) HIIT→RT groups. (**c**) Schematic illustration for the determination of skeletal muscle capillarization indices.

All muscle biopsy analyses were performed by a blinded investigator. The following indices were determined (Fig. 1): the number of capillaries surrounding a fiber (capillary contacts), the number of fibers sharing each capillary (sharing factor), and the individual capillary-to-fiber ratio, defined as the number of capillaries divided by their sharing factor, *i.e*. the number of full capillaries per individual fiber^6^. To examine the potential for blood-tissue exchange, the capillary density and the capillary-to-fiber perimeter exchange index^17^ were calculated. Fiber CSA and perimeter were assessed by automatic calculation of the software after fully encircling the borders of individual muscle fibers. Fiber circularity was calculated using the formula (4π⋅CSA)/(perimeter)^2^, and only fibers with a circularity higher than 0.65 were considered for analysis (perfect circle = 1.0). On average, 68 ± 15 (week 0), 75 ± 20 (week 12), and 74 ± 18 (week 24) fibers were analyzed per biopsy sample for the overall capillary-to-fiber ratio. For all further capillarization indices, CSA, and perimeter, 62 ± 12 (week 0), 66 ± 17 (week 12), and 67 ± 11 (week 24) fibers were analyzed per biopsy sample. The staining protocol for SDH activity resulted in muscle fibers rich in SDH staining dark blue and densely granulated, and muscle fibers poor in SDH staining light blue and sparsely granulated. Quantification was performed by classifying fibers of a given section according to their relative staining intensity as fibers showing high (darkest staining), weak (intermediate staining) or no (weakest/no staining) SDH activity. All fibers on a given subset image showing distinct staining and limited signs of damage were considered for quantification resulting in 291 ± 144 (week 0), 260 ± 78 (week 12), and 335 ± 130 (week 24) fibers that were considered for analysis.

### Sample size and statistical analyses

The necessary sample size was estimated to be 12 participants per group at a level of significance of 5% and a statistical power of 80%. However, only a total of 20 participants could be recruited of which two withdrew from the study for personal reasons and one had to be excluded because he did not meet the training frequency guidelines. Data are presented as mean values ± standard deviations (SD). Normality of data was ascertained by a Shapiro-Wilk test. Baseline values of the two groups were compared with an independent sample *t*-test. A paired sample *t*-test was performed to detect changes during the habitual course observation phase. A univariate general linear model was applied to examine the changes that occurred during the RT and HIIT phase. For this analysis, the differences post-pre (Δ) of each variable was compared between groups. Significant differences within groups from pre- to post-phase were assessed by parameter estimates. The effect of the preceding habitual course observation phase or HIIT on RT adaptation was analyzed with ANCOVA. We analyzed the differences between the groups by using the Δ parameter of phase 1 (habitual course observation phase or HIIT) as a covariate to account for the alterations that occured during phase 1. The level of significance was set at *P* < 0.05. The effect size was described as *η*^2^_p_ (partial eta-squared). All statistical analyses were performed using the software SPSS Statistics 24.0 (SPSS, Chicago, IL, USA).

### Compliance with ethical standards

#### Ethical approval

All procedures in the study involving human participants were in accordance with the ethical standards of the institutional research committee and with the 1964 Helsinki declaration and its later amendments.

### Informed consent

Informed consent was obtained from all individual participants included in the study.

## Results

### Participant’s characteristics and habitual course observation phase

Participants of the two groups did not differ in physical characteristics and functional measures at baseline (Table 1). During the habitual course observation phase, all capillarization indices remained constant (Table 2). Furthermore, there was no alteration in overall CSA and overall muscle fiber perimeter (Table 2). The proportion of muscle fibers displaying high, weak, and no SDH activity also remained unaltered (Table 3).

**Table 1:** Participants’ characteristics and functional measures at baseline for two groups with different exercise intervention protocols.

	HC→RE (*n* = 8)	HIIT→RE (*n* = 9)
Age (years)	66.5 ± 2.9	67.1 ± 4.4
Body mass (kg)	79.0 ± 9.6	84.6 ± 16.3
Height (cm)	175.6 ± 4.7	178.9 ± 5.6
BMI (kg·m^−2^)	26.0 ± 3.2	26.3 ± 4.2
*P*_peak_ (W)	178.8 ± 35.2	193.3 ± 24.3

Data are mean ± SD. Abbreviations: BMI, body mass index; $\dot{V}{O}_{2peak}$, peak oxygen uptake; *P*_peak_, peak power.

**Table 2:** Results of the habitual course observation phase.

	week 0	week 12
Overall capillary-to-fiber ratio	1.77 ± 0.30	1.78 ± 0.34
Capillary contacts	5.12 ± 0.70	5.25 ± 0.80
Sharing factor	2.75 ± 0.11	2.75 ± 0.10
Individual capillary-to-fiber ratio	1.91 ± 0.34	1.94 ± 0.35
Capillary density (capillaries·mm^−2^)	384 ± 40	401 ± 47
Capillary-to-fiber perimeter exchange index (capillaries·1000 µm^−1^)	6.44 ± 0.73	6.59 ± 0.85
Cross-sectional area (µm^2^)	5117 ± 853	5024 ± 711
Perimeter (µm)	295 ± 26	294 ± 19

Data are mean ± SD for *n* = 8 older men.

**Table 3:** Succinate dehydrogenase (SDH) activity in both training groups at the three points in time.

	HC→RT (*n* = 8)			HIIT→RT (*n* = 9)		
	week 0	week 12	week 24	week 0	week 12	week 24
SDH high activity	28.1 ± 7.9%	27.4 ± 6.9%^###^	40.8 ± 7.4%***^,#^	29.8 ± 7.0%	38.2 ± 6.4%***^,###^	39.4 ± 5.5%*^,#^
*η*^2^_p_		0.020/0.652	0.804/0.390		0.773/0.652	0.008/0.390
SDH weak activity	39.3 ± 7.3%	40.3 ± 5.9%	36.2 ± 5.0%	38.0 ± 4.9%	40.6 ± 6.5%	40.9 ± 3.8%
*η*^2^_p_		0.011/0.019	0.179/0.170		0.089/0.019	0.026/0.170
SDH no activity	32.5 ± 7.3%	32.3 ± 6.1%^##^	23.0 ± 3.6%**	32.3 ± 8.1%	21.2 ± 7.2%**^,##^	19.7 ± 5.9%
*η*^2^_p_		0.001/0.538	0.549/0.072		0.721/0.538	0.084/0.072

Data are mean ± SD. HC→RT, habitual course observation phase (HC, 12 weeks) followed by resistance training (RT, 12 weeks); HIIT→RT, consecutive training regimens of high-intensity interval training (HIIT, 12 weeks) followed by RT (12 weeks). *P < 0.05, **P < 0.01, ***P < 0.001, significant differences versus previous point in time within group; ^#^*P* < 0.05, ^##^*P* < 0.01, ^###^*P* < 0.001, significant group x time interaction for previous phase. *η*^2^_p_ is displayed for within group comparisons before slash and for group x time interactions after slash.

### Differences between RT and HIIT

RT and HIIT resulted in significant increases in most capillarization indices compared to pre-training values (Table 4). There were no significant differences between single periods of RT and HIIT in the increases in overall capillary-to-fiber ratio (*P* = 0.517, *η*^2^_p_ = 0.029) and capillary contacts (*P* = 0.659, *η*^2^_p_ = 0.013), in the decrease in the sharing factor (*P* = 0.510, *η*^2^_p_ = 0.029), and in the increase in the individual capillary-to-fiber ratio (*P* = 0.614, *η*^2^_p_ = 0.017). Capillary density remained constant during both training interventions, without a difference between RT and HIIT (*P* = 0.208, *η*^2^_p_ = 0.103). Capillary-to-fiber perimeter exchange index was solely increased through HIIT, but there was no significant group by time interaction (*P* = 0.136, *η*^2^_p_ = 0.142). Overall CSA was significantly increased by RT (pre-training: 5024 ± 711 µm^2^, post-training: 5911 ± 839 µm^2^, *P* = 0.040, *η*^2^_p_ = 0.251), while it was not significantly altered during HIIT (pre-training: 5052 ± 1576 µm^2^, post-training: 5666 ± 1022 µm^2^, *P* = 0.120, *η*^2^_p_ = 0.153). The proportion of muscle fibers with high SDH activity increased significantly by RT and HIIT (Table 3), whereas the proportion of muscle fibers with no SDH activity was significantly reduced by both training modalities. There were no alterations in the proportion of muscle fibers with weak SDH activity during the whole intervention phase (Table 3).

**Table 4:** Capillarization indices before and after 12 weeks of resistance training (RT) or high-intensity interval training (HIIT).

	RT (*n* = 8)			HIIT (*n* = 9)		
	pre-training	post-training	*η*^2^_p_	pre-training	post-training	*η*^2^_p_
Overall capillary-to-fiber ratio	1.78 ± 0.34	1.96 ± 0.26**	0.428	1.59 ± 0.30	1.82 ± 0.20***	0.577
Capillary contacts	5.25 ± 0.80	5.79 ± 0.63**	0.452	4.70 ± 0.84	5.33 ± 0.49**	0.562
Sharing factor	2.75 ± 0.10	2.71 ± 0.08*	0.262	2.80 ± 0.11	2.73 ± 0.09**	0.448
Individual capillary-to-fiber ratio	1.94 ± 0.35	2.15 ± 0.30**	0.414	1.73 ± 0.36	1.97 ± 0.22**	0.541
Capillary density (capillaries·mm^−2^)	401 ± 47	374 ± 32	0.139	360 ± 43	365 ± 40	0.005
Capillary-to-fiber perimeter exchange index (capillaries·1000 µm^−1^)	6.59 ± 0.85	6.72 ± 0.55	0.034	5.84 ± 0.49	6.35 ± 0.53**	0.385

Data are mean ± SD. *P < 0.05, **P < 0.01, ***P < 0.001, significantly different to pre-training within group. *η*^2^_p_ is displayed for the within group differences from pre- to post-training.

### Differences between RT without prior HIIT or subsequent to HIIT

**Figure 2:**
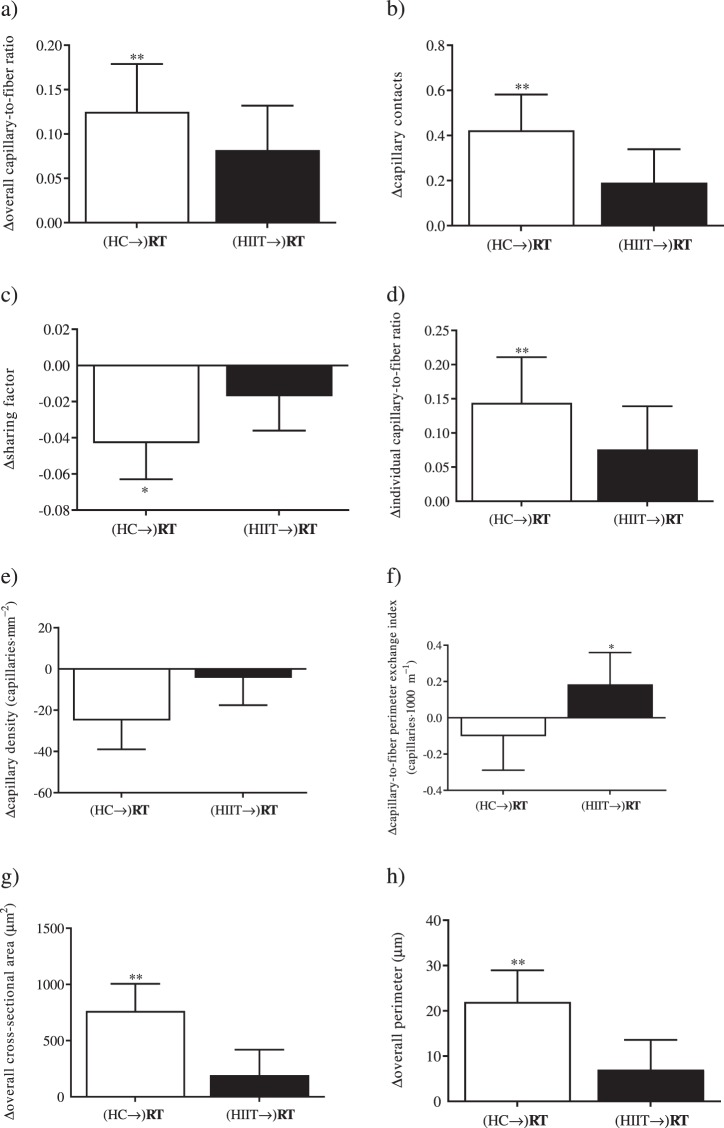
Resistance training-induced changes in the HC→RT (white bars) and HIIT→RT (black bars) groups for (**a**) overall capillary-to-fiber ratio, (**b**) capillary contacts, (**c**) sharing factor, (**d**) individual capillary-to-fiber ratio, (**e**) capillary density, (**f**) capillary-to-fiber perimeter exchange index, (**g**) overall cross-sectional area, (**h**) overall perimeter. Bars and error bars represent mean and standard deviations. *P < 0.05, **P < 0.01, significant change within group in the training period.

Most capillarization indices (Fig. 2a–e), CSA (Fig. 2g), and fiber perimeter (Fig. 2h) remained constant during RT with prior HIIT. Capillary-to-fiber perimeter exchange index increased significantly during RT with prior HIIT (Fig. 2f). There were no significant group x time interactions between RT performed with or without prior HIIT for overall capillary-to-fiber ratio (*P* = 0.618; *η*^2^_p_ = 0.022), capillary contacts (*P* = 0.350; *η*^2^_p_ = 0.084), sharing factor (*P* = 0.322; *η*^2^_p_ = 0.051), individual capillary-to-fiber ratio (*P* = 0.521; *η*^2^_p_ = 0.042), capillary density (*P* = 0.306; *η*^2^_p_ = 0.061), capillary-to-fiber perimeter exchange index (*P* = 0.314; *η*^2^_p_ = 0.051), CSA (*P* = 0.114; *η*^2^_p_ = 0.174), and fiber perimeter (*P* = 0.150; *η*^2^_p_ = 0.141). The proportion of muscle fibers displaying high (*P* = 0.741; *η*^2^_p_ = 0.008), weak (*P* = 0.554; *η*^2^_p_ = 0.026), and no SDH activity (*P* = 0.277; *η*^2^_p_ = 0.084) remained constant during RT with prior HIIT.

### Harms or unintended effects

None of the participants did experience harms or unintended effects during the study, neither due to the training interventions nor due to the muscle biopsy sampling.

## Discussion

In the present study, we found that single periods of exclusive RT or HIIT caused a similar increase of determinants for capillarization and SDH activity in older men. In addition, improved capillarization indices and SDH activity after 12 weeks of HIIT were maintained during subsequent 12 weeks of RT.

RT and HIIT without prior training resulted in similar increases in overall capillary-to-fiber ratio, capillary contacts, and individual capillary-to-fiber ratio. These results suggest that both training modalities were effective in inducing angiogenesis. The increase in capillaries per fiber was associated with a similar decrease in the sharing factor in both training groups. Before the training interventions, the majority of capillaries was positioned at the junction of three individual muscle fibers. During the training intervention, novel capillaries were primarily built alongside the border of only two muscle fibers. Hence, new capillaries were mainly formed at positions at which they shared a minimum of muscle fibers. This leads to an optimized exchange surface and a higher capacity for blood-tissue exchange. However, capillary density was not significantly altered following the intervention period of both training modalities. The significant increase of CSA in the RT group and the slight non-significant increase of CSA in the HIIT group might explain the constant capillary densities. In contrast, capillary-to-fiber perimeter exchange index was increased during HIIT. This was not the case following RT, as the significant increase in overall muscle fiber perimeter by RT prevented a significant increase in capillary-to-fiber perimeter exchange index. Important to note, the HIIT→RT group had a markedly lower baseline value for the capillary-to-fiber perimeter exchange index compared to the HC→RT group, and even the significant increase in the capillary-to-fiber perimeter exchange index during HIIT was not sufficient to reach the values of the HC→RT group. Moreover, during the second phase of the study, the RT period following the HIIT period, resulted again in a significant increase in the capillary-to-fiber perimeter exchange index, indicating that RT can in fact elevate this index. Thus, the observed difference in the adaptation of the capillary-to-fiber perimeter exchange index between HIIT and RT without prior training seems to be explained by uneven, though not significant, baseline values, and not necessarily because there is an actual difference in the underlying adaptation mechanisms between HIIT and RT. The changes in SDH activity observed during HIIT and RT without prior training further support the notion that the two training modalities share similarities in their mechanisms of adaptation. Following both types of training, the percentage of muscle fibers with high SDH activity was increased, indicating that oxidative energy metabolism was improved with both HIIT and RT. Hence, differences in oxygen flux in response to the distinct training modalities, as well as a specific adaptation of the capillary-to-fiber perimeter exchange index to HIIT seems unlikely.

Additional supportive evidence for similar adaptations in HIIT and RT arises from the results of the sequential training design. The increased capillarization indices and the improvements in oxidative enzyme activity achieved with HIIT were both preserved during 12 weeks of subsequent RT. Moreover, the capillary-to-fiber perimeter exchange index was even further increased during subsequent RT, and relative values for all other variables pointed in the same direction as in the previous HIIT phase. While it is known that capillary density can be maintained during short periods of detraining^18^, oxidative enzyme activity returns to baseline levels after only 2 weeks of detraining following an 8-week training program^19^. Specifically, oxidative enzyme activity follows an initial large decrease after training cessation and stabilizes thereafter at a level higher than prior to training^20^. Thus, our results demonstrate that RT is a sufficient stimulus to maintain or even improve HIIT-induced adaptations in capillarization indices and oxidative enzyme activity. However, because of the impossibility to allocate a specific identity to individual capillaries with the techniques applicable to human biopsies, it is not possible to determine whether the capillaries counted after RT are the same as built during prior HIIT, *i.e*. whether single capillaries or the total number could in fact be maintained. Hence, if the mechanisms underlying capillary formation are different between HIIT and RT, it is conceivable that the capillaries formed during HIIT were deconditioned after HIIT cessation and replaced by *de novo* formation via different mechanisms induced by the RT stimulus. Despite these potential limitations for interpretation, our data does not suggest that the two training modalities have strong additive effects on vascularization. This is in accordance with the only other study that used a sequential training design, wherein the reverse order of the same two training modalities, *i.e*. RT→ET, was investigated^6^. In that study, individual capillary-to-fiber ratio and capillary contacts increased during RT and were not further improved by a subsequent period of ET. Interestingly, however, capillary-to-fiber perimeter exchange index increased during RT and was further elevated during the following period of ET^6^. The further increase in capillary-to-fiber perimeter exchange index during ET may be explained by the significant decrease in muscle fiber perimeter that occurred coincidently^6^.

The increased capillarization after both, 12 weeks of HIIT or 12 weeks of RT has at least three functional benefits for older men. First, enhanced capillarization allows for an improved blood-tissue exchange and may result in greater glucose delivery and metabolization in skeletal muscle. This is consistent with previous observations revealing a favorable association between muscle capillarization and insulin sensitivity in older adults^18,21–23^. Moreover, the increase in capillary density has previously been shown to significantly correlate with higher insulin sensitivity in older adults following 6 months of ET^17^. Second, enhanced capillarization might increase leg blood flow in response to exercise, which is supported by a previous study showing increased leg blood flow and leg vascular conductance in response to exercise and feeding after 20 weeks of RT in older adults^24^. An increased femoral artery blood flow has the potential to attenuate muscle protein breakdown following exercise and feeding and might turn net protein balance into positive numbers^25^. Furthermore, the number of muscle fiber capillary contacts has been shown to correlate with myofibrillar protein synthetic response to a mixed meal^26^. Third, it has been proposed that blood supply is a critical factor for the maintenance or gain of muscle mass in young^27^ and older adults^8,9^ in response to RT. Taken together, based on the beneficial effects of improved capillarization listed above, and in conjunction with our own findings, both HIIT and RT appear to be effective countermeasures to mitigate age-related metabolic impairments and decreases in skeletal muscle mass with increasing age.

Our findings are supported by previously published data, showing that constant moderate intensity ET is an adequate stimulus to substantially induce angiogenic adaptations in older adults^6,7,11^. We extend the scientific knowledge, showing that a HIIT protocol improved capillarization indices in older men. Furthermore, we observed a significant decrease in the sharing factor, which has previously been reported to be non-significantly lowered following constant moderate intensity ET^6,7^. Contrary to the uniform findings on the effects of ET on capillarization, previous studies on the effects of RT on capillarization indices were less consistent. Our results are in line with a few studies that reported an increase in capillarization indices after 9-12 weeks of RT^6,9,12^, but are in contrast to other studies that showed no significant increases after 12-24 weeks of RT^8,13^. Unfortunately, it is not possible to identify the reasons for this discrepancy, because the RT protocols of these studies do not provide sufficient details for comparisons. While information about exercise types, sets, repetitions, and training load is provided, details on other mechano-biological descriptors of resistance exercise stimuli^28^ are lacking. These descriptors, including the fractional and temporal distribution of muscle actions per repetition, the duration of one repetition, and the time under tension (TUT), are substantially determining the character of the applied stimulus, which in turn affects the molecular patterns induced in exercised muscle fibers. In particular, TUT (of one single set and/or summed up for a given muscle over a complete training session) may play a pivotal role in stimulating capillarization with RT, and could have decisively varied between studies. Performing a RT exercise with a prolonged TUT and as close as possible to volitional muscular failure (as it was the case with our RT protocol), may increase hypoxic, mechanical and metabolic stress, and thereby stimulate angiogenesis in a sustained manner. In contrast, when TUT is too short and/or the exercise is not performed to volitional muscular failure, the stresses might be lower and thus the angiogenic stimulus weaker. Moreover, prolonged TUT may be necessary to recruit and fatigue high threshold motor units, and thereby to stimulate angiogenesis in fast (*i.e*. MyHC-2) muscle fibers more effectively. Nevertheless, to draw firm conclusions regarding the role of TUT or other mechano-biological descriptors, further studies are required, which vary these factors and directly compare their effects on capillarization.

### Limitations of the study

We have chosen a parallel group study design, since this represents the most feasible setting with human participants. The most accurate study design to compare the training effects of both training modalities and their sequential application is a randomized crossover design. However, according to current knowledge on the persistence of muscular adaptations in conjunction with skeletal muscle memory, the use of this study design is not appropriate for human participants. While muscle memory in response to ET was questioned recently^29^, facilitated mitochondrial remodelling was observed after RT^30^ and training-induced increases in the number of myonuclei are long-lasting, resulting in an altered precondition for future investigations^31^. In previous studies, it has been proposed that the lifespan of human myonuclei is at least 15 years^31,32^. Consequently, the notion of a washout period between the two arms of the crossover design and its application in human participants is at least questionable.

A further limitation of the current study is that only male participants have been investigated. Recent research has suggested that sex may affect the acute exercise response and/or long-term exercise adaptation including the vascular system^33,34^. Therefore, our results do not necessarily apply for both sexes of the elderly population and more studies are required to clarify this.

## Conclusion

A 12-week period of exclusive RT without prior training resulted in similar improvements in most capillarization indices and SDH activity as compared to 12 weeks of exclusive HIIT. Furthermore, the HIIT-induced improvements in capillarization and oxidative enzyme activity were maintained during a subsequent 12-week RT intervention. Hence, the applied RT protocol represents a sufficient stimulus to preserve the HIIT-induced increase in capillarization indices. Collectively, our study provides evidence that both RT and HIIT can be valid training modalities to induce angiogenesis and improve muscle fiber capillarization indices in older men. These adaptations may help to prevent the development of sarcopenia and metabolic impairments with increasing age.
